# Evaluation of YouTube Video Content Related to the Management of Hypoglycemia

**DOI:** 10.7759/cureus.12525

**Published:** 2021-01-06

**Authors:** Ayse Cetin

**Affiliations:** 1 Emergency Medicine, Liv Hospital, Istanbul, TUR

**Keywords:** hypoglycemia, emergency department, youtube, discern, gqs

## Abstract

Objective

Hypoglycemia is a syndrome characterized by decreased blood glucose levels and can result in morbidity and mortality. Often, patients seek information about hypoglycemia from the Internet and especially YouTube. The objective of this study was to investigate the quality and reliability of the 50 most viewed videos related to hypoglycemia on YouTube.

Methods

The links of 50 videos that met the criteria were copied to spreadsheet software. Attributes of the uploaders, the content of the videos, their screening time, the date they were uploaded, the number of days since the upload date, the number of daily views, comments, likes, dislike, and video power indexes were recorded. The videos were assessed by two independent emergency specialists using DISCERN and global quality scales (GQS).

Results

Of the selection, 27 (54%) videos were uploaded by health channels, 11 (22%) by physicians, nine (18%) by hospital channels, and three (6%) by patients. Furthermore, 30 (60%) of these 50 videos were real-content videos and 20 (40%) were animations. The average DISCERN score given by the researchers to 50 videos was 3.72 ± 0.90 (min-max: 1-5) and the average GQS score was 3.65 ± 0.88. Mean video power index (VPI) value was determined as 92.64 ± 11. According to Pearson’s analysis, there was a good agreement between the two observers.

Conclusion

Videos uploaded by physicians were useful. However, health videos should only be uploaded by physicians. Uploading videos with content that the patients and their relatives can clearly understand is of paramount importance.

## Introduction

Hypoglycemia is a syndrome that occurs with decreasing blood glucose levels and can result in morbidity and mortality in the person. It is one of the common causes of admission to the emergency department, and can also lead to a spectrum ranging from a slight loss of concentration to loss of consciousness and death as well as cause a wide variety of clinical symptoms [1]. It is known that a large proportion of patients worldwide admitted to the emergency department and diagnosed with hypoglycemia are diabetic patients. Insulin used in the treatment of diabetes is among the common causes of hypoglycemia [2].

Currently, there is no definitive treatment for diabetes and these patients receive lifelong treatment. Therefore, both patients and patient relatives seek information from different sources. One of the main sources of information is the Internet. Some previous studies reported that 80% of patients or their relatives were looking for information about their illness on the Internet [3,4].

YouTube is one of the most common resources where patients go to seek information on the Internet. According to the statistics of YouTube for 2020, it has 2 billion users worldwide. It has been reported that one billion hours of video are watched daily on YouTube and 400 hours of video are uploaded every minute worldwide [5] and is one of the most visited social media platforms by professionals and individuals alike due to its free access [6]. However, the scientific validity and reliability of health videos uploaded to YouTube are still controversial [7-8]. Most of the content uploaded by non-professional persons and/or organizations can be misleading and contain information without scientific grounds. In addition, there is no guarantee that the videos uploaded by individuals and/or organizations in the health field are of absolute quality, reliable, and sufficient. However, studies on this subject in the literature indicate that patients believe that the information they obtain on the Internet is at least as reliable as the doctors' [9]. Many previous studies have reported that the quality of health-related videos on YouTube is low or medium quality [10-12]. These results show that health-related videos on YouTube need to be supervised.

In this study, we aimed to investigate the quality and reliability of the 50 most relevant and most viewed videos with hypoglycemia content on YouTube.

## Materials and methods

Collection of data

The terms "diabetic emergency," "hypoglycemia," "hypoglycemia emergency," and "diabetes management emergency," which were determined by consensus by two emergency medicine specialists, were entered on YouTube separately and "relevance" was selected from the filtering options and the videos listed as available on November 1, 2020. Among the listed videos, those with advertising content, less than 60 seconds long, more than 60 minutes long, in a language other than English, and videos with repetitive content were excluded. The links of 50 videos that meet the criteria have been copied and transferred to a Microsoft Excel spreadsheet. The attributes of the uploaders, the content of the videos, their screening time, the date they were uploaded, the number of days since the upload date, the number of daily views, comments, likes, dislikes, and video power indexes were recorded. Daily views of videos were calculated according to the formula (date viewed for the study - date uploaded / total number of likes).

Video power index value is calculated according to the following formula: VPI = like count / (like count + dislike count) x 100.

Data analysis

Fifty videos included in the study were evaluated separately by two emergency medicine specialists and were scored separately according to quality criteria for consumer health information (DISCERN) and global quality scale (GQS), which were used in many studies before [13-15].

DISCERN

**Figure 1:**
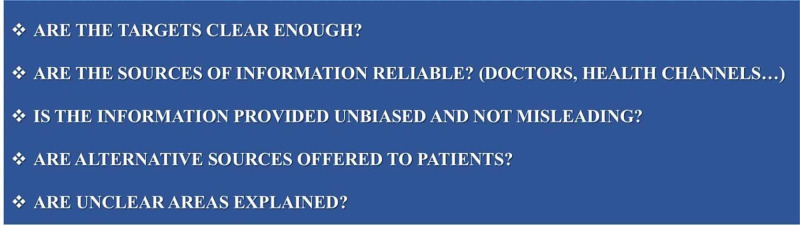
Questions used in video evaluation with the DISCERN scale

The DISCERN scale structured by Singh et al. was used to evaluate the reliability of the 50 videos analyzed [15]. The DISCERN scale consists of five questions in total, and each question is answered yes or no. Yes answer is 1 point and no answer is 0, and a maximum of 5 points can be obtained. High scores from the scale indicate the reliability of the video content [16]. Questions on the DISCERN scale are given in Figure 1.

GQS scale

**Figure 2:**
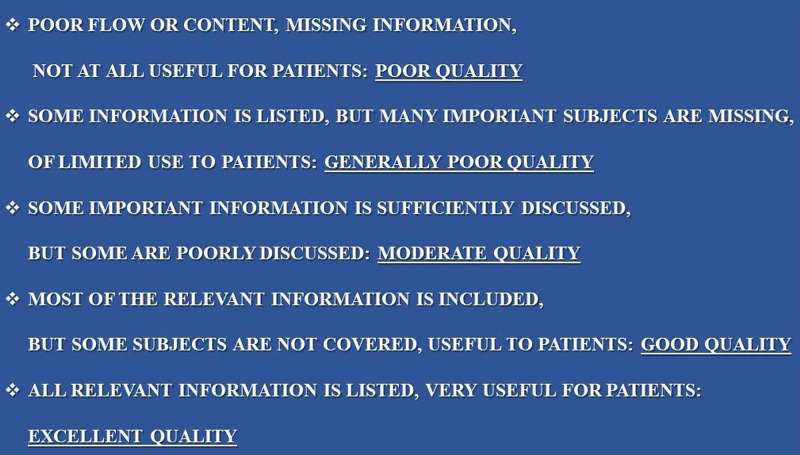
Questions used in video evaluation with the GQS scale

In the GQS scale developed by Bernard et al., points are awarded after the Web content is completely evaluated. In the GQS scale, the quality of the examined content is evaluated with a five-point system [17]. According to the GQS scale, information accessibility, quality, general flow, and how the evaluator thinks the content will be useful to the individual are evaluated. One point from the scale indicates low quality, two points for low quality or limited use, three points for somewhat useful, four points for useful, and five points for useful/excellent quality [17]. Questions on the GQS scale are given in Figure 2. Here, 1-2 points from the GQS and DISCERN scales indicate that the video content is misleading, 3 points indicate that it is of medium quality and reliability, and 4-5 points indicate that the video content is beneficial for the viewers.

Statistical analysis

Data entry and analysis were performed using SPSS 23.0 statistical software (Statistical Package for Social Sciences, IBM, Armonk, NJ). Continuous variables were expressed as mean ± standard deviation, minimum and maximum descriptive statistics, while nominal variables were given as frequency and percentage. Continuous variables were compared with independent sample t-test, and nominal variables with chi-square test. The correlation between two rates was examined by Spearman’s correlation analysis and the agreement between them was evaluated using alpha coefficients. P<0.05 values were considered statistically significant.

## Results

Of the 50 videos included in the study, 27 (54%) were uploaded by health channels, 11 (22%) by physicians, nine (18%) by hospital channels, and three (6%) by patients. While 30 (60%) of these 50 videos were real content videos, 20 (40%) were animated.

The length of the videos, the number of views, the time since the upload date, the number of daily views, the number of comments, likes and dislikes are given in Table 1.

**Table 1:** General characteristics of the videos

	Mean ± SD	Median	Min	Max
Video Length (seconds)	561.32 ± 387.71	400	60	1400
Number of Views (total)	148.268 ± 159542.4	30.339	1.000	773.000
Time since Video Upload (days)	1424.52 ± 919.92	1184	115	3626
Number of Views (daily)	91.04 ± 148.59	280	2	755
Comments (count)	66 ± 132	13	0	755
Likes (count)	935 ± 1736	58	1	9400
Dislike (count)	31.8 ± 53	21	0	272

The nature of the uploaders of the videos included in the study, the content of the videos and the number of views are given in Table 2.

**Table 2:** Distribution of video content according to the nature of the uploaders and the number of views

	General Information	Education	First Aid	Patient Experience	Surgery	Views
Health Channel	4	11	9	0	2	2.687.269
Hospital Channel	0	5	2	2	0	579.793
Physician	1	8	2	0	0	1.445.597
Patient	0	0	0	3	0	95.044

The total number of views of 50 videos included in the study was found to be 4.997.792.

The number of views of the most watched 10 videos, the quality of the uploaders, the video content, the upload date and VPI values are given in Table 3.

**Table 3:** General characteristics and VPI values of the 10 most watched videos

Views	Uploader	Content	Date	VPI (%)
773.000	Health Channel	First Aid	2012	100
275.000	Health Channel	First Aid	2013	98.66
712.606	Physician	Education	2013	95.73
374.560	Health Channel	Education	2014	92.36
226.094	Patient	Patient Experience	2016	97.78
172.000	Health Channel	Genel Bilgi	2017	97.08
173.073	Health Channel	First Aid	2017	97.08
276.355	Physician	Education	2017	97.92
173.077	Health Channel	First Aid	2017	97.08
329.000	Physician	First Aid	2019	99.19

The average DISCERN score given by the researchers to 50 videos was 3.72 ± 0.90 (min-max: 1-5) and the average GQS score was 3.65 ± 0.88. Mean VPI value was determined as 92.64 ± 11. The average DISCERN score given by the researchers to animated videos was 3.82 ± 0.92 (min-max = 3-5) and the average GQS score was 3.85 ± 0.92. The average DISCERN score given to real content videos was 3.63 ± 0.96 (min-max: 2-5) and the average GQS score was 3.6 ± 0.92 (min-max: 1-5).

The average DISCERN score given by the first researcher to the videos was 3.84 ± 0.85 (min-max: 2-5) and the average GQS score was 3.74 ± 0.86 (min-max: 1-5).

The average DISCERN score given by the second researcher to 50 videos was 3.56 ± 0.96 (min-max: 1-5) and the average GQS score was 3.64 ± 0.91 (min-max: 1-5). Accordingly, a good level of consistency was found between the first and the second observer in terms of both DISCERN (r = 0.819, p <0.001) and GQS scores (r = 8.344, p <0.001). Average DISCERN, GQS and VPI values according to the nature of the uploaders are given in Table 4.

**Table 4:** DISCERN, GQS and VPI values according to the nature of the uploaders

Uploaders	Count	DISCERN	GQS	VPI (%)
Health Channel	27	3.59 ± 0.87	3.42 ± 0.86	92.53 ± 10.71
Hospital Channel	9	3.55 ± 0.91	3.83 ± 0.89	93.38 ± 10.80
Physician	11	4.68 ± 0.96	4.77 ± 0.96	95.11 ± 11.25
Patient	3	1.5 ± 1.07	1.66 ± 1.01	82.26 ± 13.53

In our study, it was determined that three videos got 1-2 points and had misleading content and all of these videos were uploaded by the patients. While we found that the videos were generally of medium quality, videos uploaded by physicians had useful and valid content (Table 5).

**Table 5:** Quality of videos based on uploaders' attributes

Uploaders	Misleading	Medium Quality	Useful	Total
Health Channel	0	25	2	27
Hospital Channel	0	6	3	9
Physician	0	3	11	11
Patient	3	0	0	3

When the videos were generally examined, it was found that three (6%) videos included misleading content, 31 (62%) videos had medium-quality content, and 16 (32%) videos had helpful and reliable content.

## Discussion

Hypoglycemia is an endocrine emergency that can alter the mental state of the patient, and may result in lethargy, confusion, and organ dysfunction. Hypoglycemia is defined as a serum glucose level of <50 mg/dL in men, <45 mg/dL in women and <40 mg/dL in children. In a retrospective study conducted by Lipska et al., It was shown that hospital admissions due to hypoglycemia increased from 94 to 105 per 100,000 people between 1999-2011 [18]. Severe hypoglycemia is a medical emergency and is very important for patients with diabetes. Hypoglycemia is a major complication of insulin therapy, causing approximately 100,000 emergency room admissions per year in the USA [19]. Hypoglycemia has been associated with increased mortality rates in hospitalized patients, and this is due to the nature of the disease in patients susceptible to be hypoglycemic [20]. Patients who have a hypoglycemic attack leading to unconsciousness should be taken to the nearest emergency room without wasting time. From this point of view, patients susceptible to hypoglycemia try to obtain information about their disease from any possible source. For this reason, as with many diseases, hypoglycemic patients most commonly use the Internet and especially the YouTube platform to obtain information about their diseases. However, the quality and reliability of the information available on these platforms can be controversial and many studies have been conducted on this subject [20-23]. In these studies, content from videos were analyzed using various rating scales.

DISCERN [15,23] and GQS [13,24] scales were widely used to measure the quality and reliability of the videos in the studies, both of which we also utilized in this paper. 

In our study, we found that the videos were generally of medium quality. In most of the studies previously reported in the literature, it was stated that the videos were poor or moderate [25,26]. Researchers accredited this to the nature of the uploaders of the videos. It has been reported that the videos uploaded by patients and their relatives are bad and misleading [27]. We found that only three of the 50 videos we examined were uploaded by patients and all of these contained misleading content. However, it was also reported that videos uploaded by patients were viewed and liked more than videos uploaded by professionals in some previous studies [10,12].

There is currently no control mechanism for the content of videos uploaded to YouTube, and anyone who wishes, regardless of its nature, can upload any content to YouTube. In this context, YouTube provides advertising and promotion opportunities to individuals and organizations for commercial purposes. For this purpose, health organizations, like most organizations, advertise their own services on YouTube on various diseases and health problems. Nevertheless, since there is no audit and control mechanism for this information, there may be some doubts about the quality and reliability of this information. Despite this, patients are increasingly trying to benefit from the content on YouTube and learn about their diseases before applying to health institutions and seeking a medical solution to their problems.

In our study, we found that 27 of the 50 videos examined were uploaded by health channels, nine videos were uploaded by hospital channels and that these videos were of medium quality/reliability. The reasons for this were that the video content uploaded by health channels and hospital channels was not sufficiently up to date, the information was not given at a sufficient level, the use of medical terms that could not be understood by the viewers, and the promotion of the specific health institution. In the study conducted by Yurdaisik, it was determined that the videos uploaded by health channels were also of medium quality [10]. Similarly, in the studies conducted by Kuru et al. [12] and Aydin et al. [11], videos uploaded by health channels were reported to be of medium quality.

In our study, we found that 11 videos were uploaded directly by physicians and all of these videos had useful/safe content. It has also been reported in previous studies that the videos uploaded by physicians are of high quality and safe [28,29]. However, there are also studies reporting that the videos uploaded by the physicians are not of sufficient quality either [10,12]. When the findings of our study and the results of the previously reported studies are examined, it becomes clear that health-related videos should only be uploaded by physicians. However, we think that videos uploaded by physicians should also go through a control mechanism and be updated periodically.

Limitations of the study

This study has some limitations. Videos were viewed and evaluated as snapshots. However, as is known, the number of views, likes, dislikes, and comments of videos on YouTube can change at any time. In addition, as it is known, research results may vary according to the geographical location of the viewer. Finally, it is inevitable that the IP history of the computer performing the investigation will have an impact on the results. Nevertheless, the strength of our study is that it is the first study in the literature evaluating the quality and reliability of YouTube videos on hypoglycemia.

## Conclusions

In this study, we analyzed the most relevant and most-watched videos with hypoglycemia content on YouTube. We found that the videos uploaded by the patients were misleading, and the videos uploaded by the health and hospital channels were of medium quality and reliability. We came to the conclusion that only videos uploaded by doctors have useful content and thus health videos should only be uploaded by them. In addition, the increase in the number of videos about deadly conditions such as hypoglycemia, uploading awareness videos and uploading videos with content that the patients and their relatives can clearly understand is also of great significance.
